# A high nutrient dense diet alters hypothalamic gene expressions to influence energy intake in pigs born with low birth weight

**DOI:** 10.1038/s41598-018-23926-x

**Published:** 2018-04-03

**Authors:** Jingbo Liu, Shanchuan Cao, Ming Liu, Liang Chen, Hongfu Zhang

**Affiliations:** 1 State Key Laboratory of Animal Nutrition, Institute of Animal Sciences, Chinese Academy of Agricultural Sciences, Beijing, 100193 P. R. China; 2 School of Life Science and Engineering, Southwest University of Science and Technology, Mianyang, 621010 Sichuan P. R. China

## Abstract

The low birth weight (LBW) individual had greater risk of developing metabolic dysfunction in adulthood. The aim of this study was to test whether the LBW individual is more prone to glucose intolerance on a high nutrient dense (HND) diet, and to investigate the associated hypothalamic gene expressions using pigs as model. The intake of digestible energy intake, if calculated on a body weight basis, was greater in LBW pigs than that of normal birth weight (NBW) pigs. The LBW pigs fed the HND diet had greater digestible energy intake than those fed the NND diet at adulthood, which did not occur for NBW pigs. Notably, up-regulated hypothalamic toll-like receptor 4, interleukin 6 and phospho-NFκB p65 expressions, and the altered expressions of hypothalamic leptin receptor, suppressor of cytokine signaling 3, agouti-related protein and proopiomelanocortin predicted the overconsumption of energy intake and development of glucose intolerance in LBW pigs fed the HND diet. Collectively, pigs born with LBW had a distinct hypothalamic leptin signaling to a high nutrient dense diet, which contributed to greater energy intake and glucose intolerance.

## Introduction

Low birth weight (LBW), a consequence of insufficient nutrient transfer during the intrauterine phase, affects several important physiological processes involved in energy metabolism, cellular signaling, redox balance, and stress response in a variety of tissues including intestine, liver and muscle^1^, contributing to increased morbidity and mortality during the neonatal phase, and poor postnatal growth rates and health status throughout life^2,3^.

However, some of them could exhibit catch-up growth and compensate for their intrauterine undernutrition by special dietary regimens^4^. Chronically increased amino acids during the late phase of gestation improved insulin secretion of islet cell in growth-restricted fetal sheep^5^. When LBW piglets received a similar level of nutrition as piglets born with normal birth weight (NBW), they had equal growth performance compared with that of NBW piglets^6^. Low birth weight pigs provided with a high quality and expensive dietary regime might ensure maximum growth and increased profitability^7^. These results indicated that sufficient nutrient intake was necessary for the LBW individual to compensate for their early growth restriction. On the other side, some of the researches have revealed that low birth weight offspring had an increased risk of developing the metabolic syndrome when faced with a nutritional mismatch in postnatal life^8,9^. While the link between impaired fetal growth and the risk of developing postnatal metabolic syndromes is undoubtedly strong, the underlying mechanisms involved in this process remained ill-defined. Some of these mechanisms include epigenetics, alterations in transcription factor activity, mitochondrial dysfunction and impaired organogenesis^10,11^.

The regulation of nutrient intake and energy metabolism mainly takes place in the hypothalamus as the most interesting area due to its co-expression of orexigenic neuropeptide Y (NPY), agouti-related protein (AGRP) neurons, anorexigenic proopiomelanocortin (POMC) and cocaine- and amphetamine-regulated transcript neurons, which are key regulators of energy intake^12^. Leptin, a major adipokine mainly secreted by large adipocytes, can enter the brain and regulate those neurons and thus food intake via its binding to the long form leptin receptor (LEPR), predominantly localized in the hypothalamic ARC neurons^13^. The role of leptin signaling in the nutritional programming has received increasing interest because the central leptin signaling was found to be differentially organized by birth weight in pigs^14^ and rodent animals^15–17^, and may very probably result in early catch-up growth and the development of type 2 diabetes mellitus.

The pigs showed physiological similarity in body size, fat cell size, nutrient digestion, absorption and metabolism, which could offer several advantages in the study of cardiovascular diseases, blood dynamics, nutrition, general metabolic functions, digestive-related disorders, respiratory diseases, diabetes, kidney and bladder diseases, organ-specific toxicity, dermatology and neurological sequelae using pigs as animal model for human nutrition^18–21^. Additional, in modern swine production, cases of naturally intrauterine growth restriction (IUGR) arise in highly prolific sows due to high ovulation rates and the subsequent uterine crowding^22^, thus providing natural models for the investigation of biology in human infant who are born with IUGR. Therefore, in the present study, the LBW and NBW pigs were fed a normal- or high nutrient dense diet to test the hypothesis that whether hypothalamic genes and proteins were differentially expressed in LBW pigs to alter their postnatal energy intake level and the glucose tolerance.

## Materials and Methods

### Experimental design, diets and animals

All experimental procedures used in this study were in accordance with the National Research Council’s Guide for the Care and Use of Laboratory Animals, and were approved by the Animal Care and Use Committee of Chinese Academy of Agricultural Sciences, and followed the current laws of animal protection.

In order to obtain piglets with different birth weights, the birth weight of newborn piglets was recorded, and those with average litter birth weight were defined as NBW piglets, whereas those with birth weight two standard deviations lower than the average litter birth weight were defined as LBW piglets. The newborn piglets were allowed to suckle freely from their dams until weaning at an average age of 28 ± 2 days. Consequently, a total of 64 cross-bred castrated male Duroc × (Landrace × Yorkshire) piglets were obtained from thirty-two litters (one NBW piglet and one LBW piglet were selected per litter) in the present study. Then the pigs were fed a normal nutrient dense (NND) diet or a high nutrient dense (HND) diet by supplementing 10% soybean oil and 5% additional casein at the expense of corn starch (Table 1). Dietary nutrient composition was formulated to meet or exceed the nutrient requirement of pigs, and five-phase diets (Table 1) were formulated as recommended by the NRC^23^. The feeding experiment was started at d 28 postnatal, and lasted for 150 days. From the beginning of the experiment d 1 to d 90 of the experiment, two pigs were reared per pen, and then pigs were caged individually from d 91 to 150 of the experiment. During the entire experimental period, feed and water was fed ad libitum. The pigs were provided artificial light from 7:00 am to 19:00 pm, and the temperature of facility was controlled at 18~22 °C except for the higher temperature at 22~28 °C from d 1 to 30 of the experiment.

**Table 1:** Composition of the experimental diet (as fed).

Ingredients	7–11 kg phase		11–25 kg phase		25–50 kg phase		50–75 kg phase		75-slaughter	
	NND^b^	HND	NND	HND	NND	HND	NND	HND	NND	HND
Corn grain	421	420.1	503.3	502.3	559	558	596.8	596.5	645.3	644.2
Corn starch	150		150		150		150		150	
Soybean meal (CP 46.5%)	135	135	240	240	240	240	210	210	180	180
Soy protein concentrate (CP 65.0%)	100	100	40	40						
Porcine plasma protein (CP 70.0%)	25	25								
Fish meal (CP 60.2%)	40	40	40	40	25	25	20	20		
Whey, dehydrated	100	100								
Casein (CP 88.0%)		50		50		50		50		50
Soybean oil		100		100		100		100		100
CaCO_3_	8	9	9	9.6	9	9.5	8.5	9	7.5	9
Choline chloride	1	1	1	1	1	1	1	1	1	1
Salt	3	3	3	3	3	3	3	3	3	3
CaH_2_PO_4_	6	4	4	2.5	5	4	4	3	6	4
Vitamin and mineral premix^a^	5	5	5	5	5	5	5	5	5	5
L-Lys·HCl (78.5% purity)	3	3	2.8	2.8	2	2	1.4	1	1.9	1.9
L-Thr (98% purity)	1	1	0.8	0.8	0.5	0.5	0.3	0.3	0.3	0.4
L-Val (98% purity)	0	1.5		1.5		1.4		0.8		1
DL-Met (98.5% purity)	2	2.4	1.1	1.5	0.5	0.6		0.4		0.5
Total	1 000	1 000	1 000	1 000	1 000	1 000	1 000	1 000	1 000	1 000
Calculated digestible energy (MJ/kg)	3.45	3.99	3.4	3.94	3.39	3.92	3.39	3.93	3.39	3.93
Analyzed crude protein content (%)	21.53	25.9	21.06	25.43	17.91	22.26	14.95	20.73	14.29	18.57
Calculated calcium (%)	0.8	0.8	0.7	0.7	0.66	0.66	0.59	0.59	0.52	0.52
Calculated total phosphorus (%)	0.65	0.64	0.54	0.54	0.5	0.51	0.46	0.47	0.44	0.43
Calculated available phosphorus (%)	0.4	0.4	0.33	0.33	0.31	0.31	0.27	0.27	0.25	0.25
Calculated total lysine (%)	1.53	1.87	1.4	1.75	1.12	1.47	0.95	1.29	0.84	1.19
Calculated total methionine + cysteine (%)	0.86	1.08	0.79	0.98	0.65	0.81	0.57	0.75	0.5	0.7
Calculated total tryptophan (%)	0.25	0.32	0.25	0.32	0.2	0.28	0.19	0.26	0.16	0.23
Calculated total threonine (%)	0.96	1.15	0.87	1.07	0.72	0.91	0.64	0.84	0.56	0.74
Calculated total valine (%)	1.04	1.19	0.97	1.13	0.83	0.97	0.76	0.84	0.65	0.75

^a^Supplied (per kg diet): 12 800 IU vitamin A, 44 IU vitamin E, 2 600 IU vitamin D, 4 mg vitamin K, 2.4 mg vitamin B1, 8.8 mg riboflavin, 32 mg niacin, 4 mg pantothenic acid, 0.5 mg biotin, 2 mg folic acid, 0.05 mg vitamin B12, Zn, 90 mg; Mn, 4.0 mg; Fe, 90 mg; Cu, 6.0 mg; I, 0.2 mg; Se, 0.3 mg.

^b^NND, normal nutrient dense diet, HND, high nutrient dense diet.

### Records of growth performance

From the beginning of the experiment to d 90 of the experiment two pigs were reared in one pen, and the data of nutrient intake, bodyweight gain and feed efficiency were calculated using pen as the experimental unit. Thereafter, pigs were reared individually and data were recorded using each pig as the experimental unit. The body weight of each pig was recorded at d 30, 60, 90, 120 and 150 of the experiment, and the average daily feed intake, body weight gain and feed efficiency were calculated every 30 days.

### Glucose tolerance test

Intravenous glucose tolerance test (IGTT) for all pigs was conducted on d 30, 83 and 143 of experiment. After overnight fasting, basal blood samples were collected at 10 min before administration. A bolus of dextrose (500 g•L-1) was infused into ear venipuncture at a dose of 0.5 g•kg-1 body weight. A serial of blood samples was collected at 5, 10, 20, 30, 45, 60, 90 and 120 min post glucose infusion. Concentrations of blood glucose were measured immediately using a portable glucometer, Esprit (Bayer, Newbury, Berkshire, UK). The area under curve (AUC) of glucose concentration were calculated using Prism 6 (GraphPad Software Incorporated, La Jolla, CA, USA).

### Collection of tissue samples

Hypothalamus, liver tissues, skeletal muscle (*Longissimus* muscle) and adipose tissues (abdominal fat) were collected at d 90 (n = 8) and 150 (n = 8) of experiment in a fed state. Notably, hypothalamic tissue was collected as follows, after sacrifice by the intraperitoneal injection of 90 mg·kg^−1^ sodium thiopental, the brains were quickly removed from the skull and freed from excess tissues. The hypothalamic tissues were collected from a block of tissue bounded rostrally by the optic chiasma, caudally by the mammillary body, laterally by the hypothalamic sulci and dorsally by a cut 5 mm deep^24,25^. All tissue samples were frozen in liquid nitrogen and stored at −80 °C for further analysis.

### Measurement of metabolites and hormones

Blood of all pigs was collected at the beginning and at d 30, 60, 90, 120 and 150 of the experiment. Concentrations of triglycerides and total cholesterol were assayed with respective commercial kits (Nanjing Jiancheng Institute of Bioengineering, Jiangsu, China) using enzymatic methods by a Hitachi 7160 Automatic Biochemical Analyzer (Tokyo, Japan) according to the manufacturer’s instructions. Circulating concentrations of leptin were measured with a commercial ELISA kit (Elabscience, Wuhan, China). The sensitivity of detection was 0.168 ng/ml, and the inter-assay and intra-assay coefficients of variation for the leptin assay were 10.6% and 8.5%, respectively.

### Gene expression

Real-time quantitative PCR was used to detect the mRNA transcriptional abundance. Detailed procedures were as previously described^26^. The primers were synthesized commercially by TaKaRa Biotechnology (TaKaRa, Dalian, China). Synthesized cDNA was subjected to real-time PCR on an ABI 7500 Real-Time PCR System using SYBR Green PCR Master Mix (Applied Biosystems) to detect target gene expression. Primers used for target genes are toll-like receptor 4 (*TLR4*), forward primer TCA GTT CTC ACC TTC CTC CTG and reverse primer GTT CAT TCC TCA CCC AGT CTT C, interleukin 6 (*IL6*), forward primer GAC AAA GCC ACC ACC CCT AA and reverse primer CTC GTT CTG TGA CTG CAG CTT ATC, suppressor of cytokine signaling 3 (*SOCS3*), forward primer CAC TCT CCA GCA TCT CTG TC and reverse primer TCG TAC TGG TCC AGG AAC TC, *LEPR*, forward primer CTC TTG CCT GCT GGA GGA ACT TC and reverse primer TTC CAG TTT GCA CCT GTT TG, *POMC*, forward primer GTG GGA GAT GCC GAG ATT GT and reverse primer CTC CTC CTC CTC GCG CTT CT, *AGRP*, forward primer GCC CCA CTG AAG AAG ACA AC and reverse primer GTA CCC AGC TTG CGG CAG TA, *NPY*, forward primer ACC CTC GCC CTG TCC CTG CT and reverse primer ATG TGG TGA TGG GAA ATG AG, *18 S RNA* forward primer TCC GAC TTT CGT TCT TGA TTA ATG and reverse primer TGG ACC GGC GCA AGA C. The house-keeping gene used was 18S RNA and the relative gene expression levels were expressed as fold changes relative to average mRNA levels of genes in NBW pigs fed the NND diet^27^.

### Protein expression

Western blotting was used to detect targeted protein expression in the hypothalamic tissues. Briefly, total protein was extracted from frozen samples with lysis buffer (Beyotime Biotechnology, Jiangsu, China) supplemented with a protease inhibitor cocktail (Roche, USA). The homogenates were then centrifuged at 12 000 g for 30 min at 4 °C and the supernatant was isolated. The protein content was measured with a BCA protein assay kit (Beyotime Biotechnology, Jiangsu, China) on a plate reader. Protein lysates were separated on 10% SDS-PAGE gel after boiling at 95° C for 5 min and was then transferred to an apolyvinylidene fluoride (PVDF) membrane. The membrane was washed in Tris-buffered saline containing tween (TBST) and blocked in 1% bovine serum albumin (Beyotime Biotechnology, Jiangsu, China) in TBST at room temperature for one hour with gentle shaking. After that, the membranes were incubated overnight at 4 °C with the respective antibodies: α-tubulin (cat#4285, Cell Signaling Technology), SOCS3 (cat#ab78341, Abcam), Phospho-NFκB p65 (Ser536) (#3033, Cell Signaling Technology) and NFκB p65 (#6956, Cell Signaling Technology). The membranes were washed with TBST and incubated with the corresponding secondary antibody for 60 min at room temperature. The intensity of the bands on the blots was quantified by Image Lab statistical software (Bio-Rad Laboratories, CA, USA). The relative expression of targeted protein was normalized using α-tubulin as the internal protein and was presented as the fold change relative to the group of the NBW pigs fed the NND diet.

### Statistical analysis

During the entire period, all the pigs used in this trial kept health and none of them were culled due to illness or other management factors. From the beginning of the experiment to d 90 of the experiment two pigs were reared in one pen, and the data were analyzed using pen as the experimental unit. The bodyweight of the two pigs in each pen was quite similar between each other at d 30, 60, and 90 of the experiment, so we assumed that the pigs were under the same treatment. From d 91 to the end of experiment, the pigs were caged individually and the data were analyzed using the individual pig as the experimental unit. Data were analyzed using the MIXED procedure of SAS software (SAS Institute, Cary, NC, USA). The main effect of birth weight (NBW or LBW) and nutrient density (NND or HND) were tested using two-way ANOVA as fixed effects in the statistical model, and the interactions of birth weight × nutritional level were also considered as fixed effects. The pen (before d 90) or pig (after) was included as a random effect, respectively. All data are presented as means. The Tukey’s test was used to compare the differences between treatment groups. Differences with probabilities of *P* < 0.05 were considered as statistically significant.

## Results

### Dynamic energy consumption

**Figure 1:**
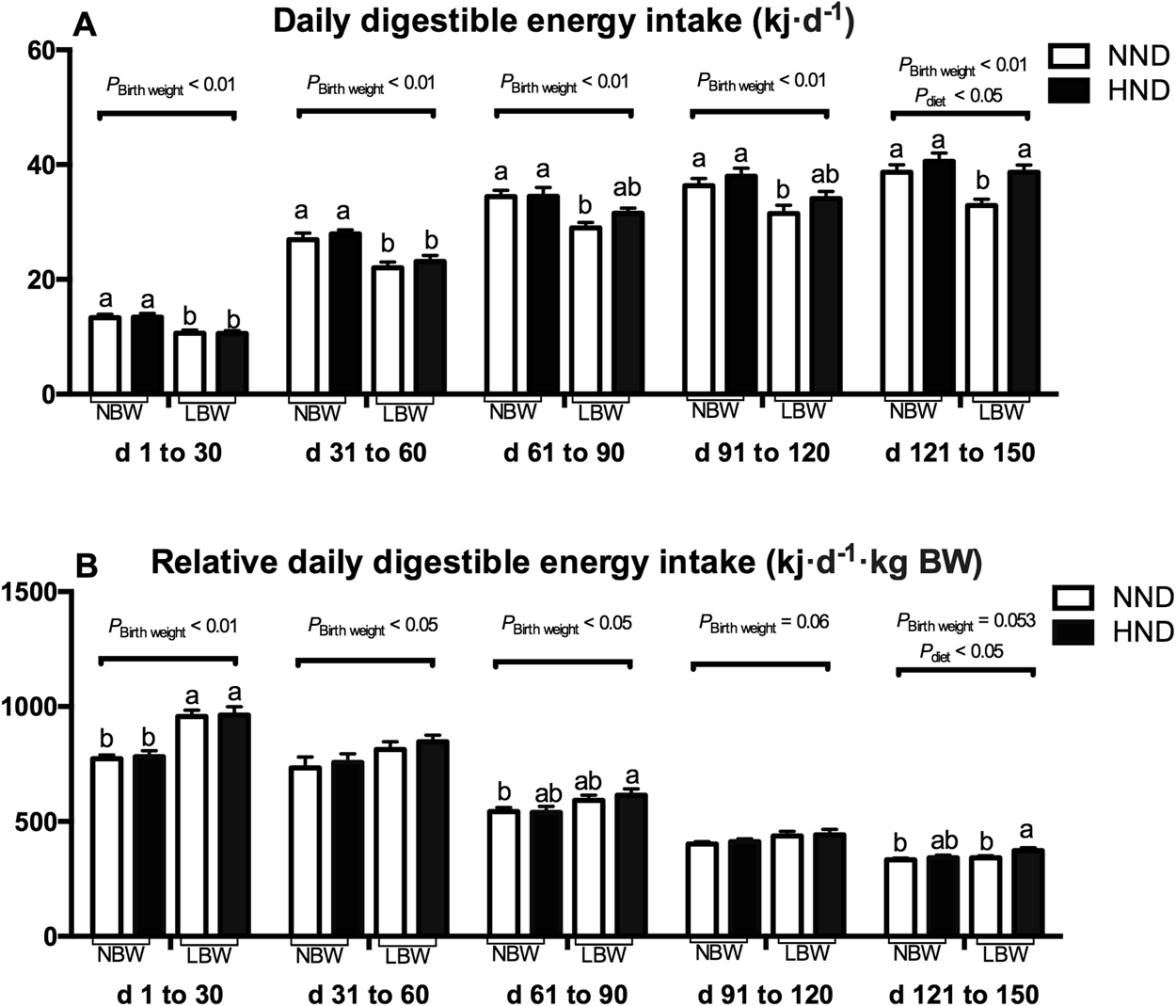
Influence of birth weight and diet on daily digestible energy intake over time (n = 8). (**A**) daily digestible energy; (**B**) relative daily digestible energy intake, daily digestible energy on a body weight basis. Column with different superscripts. ^a,b^Denotes *P* < 0.05. NBW, normal birth weight; LBW, low birth weight; NND, normal nutrient density; HND, high nutrient density.

As shown in Fig. 1A, the intake of digestible energy was significantly greater in NBW pigs compared with LBW pigs during different experimental periods (*P* < 0.01). The HND diet did not affect the intake of digestible energy from d 1 to 30, 31 to 60, 61 to 90 and 91 to 120 of the experiment, except that there was greater digestible energy intake in pigs fed the HND diet compared with pigs fed the NND diet from d 121 to 150 of experiment (*P* < 0.05). The intake of digestible energy from d 121 to 150 of the experiment was greater in LBW pigs fed the HND diet as compared with pigs fed the NND diet (*P* < 0.05), but not for NBW pigs.

The relative digestible energy intake, calculated on a bodyweight basis, was presented in Fig. 1B. Low birth weight pigs had greater relative digestible energy intake during different experiment periods (*P* < 0.05 or *P* < 0.06), and the intake of digestible energy was greater in the HND diet compared with the NND diet from d 121 to 150 of the experiment (*P* < 0.05).

### Growth traits and feed efficiency

The growth traits and feed efficiency of pigs fed different diets are shown in Table 2. The body weight of LBW pigs were lower compared with NBW pigs during the entire experimental period (*P* < 0.01). The main effect of diet or interaction between diet and birth weight did not affect the body weight (*P* > 0.05). The average daily gain was lower in LBW pigs than that of NBW pigs during the period from d 1 to 30, 31 to 60, 61 to 90 and 91 to 120 of the experiment (*P* < 0.05 or *P* < 0.01, Table 2), but were similar among different groups from d 121 to 150 of the experiment (*P* > 0.05). The feed/gain ratio was greater in LBW pigs than that in NBW pigs from d 1 to 30 of the experiment (*P* < 0.01), but not during the other periods of the experiment. The feed/gain ratio was lower in pigs fed the HND diet compared with that of pigs fed the NND diet during the period from d 31 to 60 (*P* < 0.05) and 61 to 90 (*P* < 0.01) of the experiment, but were not affected by diet in the other experimental periods.

**Table 2:** Influence of birth weight and diet on growth traits of pigs at different time of experiment.

	NBW				LBW				Significance		
	NND		HND		NND		HND		Diet	Birth weight	Interaction
	Mean	SEM	Mean	SEM	Mean	SEM	Mean	SEM			
Bodyweight, kg											
Newborn	1.42	0.11	1.43	0.12	0.93	0.10	0.92	0.09	NS	**	NS
Initial	9.66^a^	0.23	9.94^a^	0.24	6.01^b^	0.14	6.04^b^	0.13	NS	**	NS
d 30	24.75^a^	1.03	24.22^a^	1.02	16.19^b^	0.54	15.86^b^	0.59	NS	**	NS
d 60	49.62^a^	1.68	50.32^a^	1.88	38.03^b^	1.49	38.77^b^	1.52	NS	**	NS
d 90	77.40^a^	1.97	77.88^a^	1.74	60.12^b^	1.82	63.14^b^	2.08	NS	**	NS
d 120	104.73^a^	3.75	108.33^a^	3.98	83.21^b^	3.48	90.99^b^	3.70	0.11	**	NS
d 150	129.17^a^	2.93	133.09^a^	4.01	101.49^c^	4.23	116.46^b^	4.59	NS	**	NS
Average daily gain, g/d											
d 1 to 30	502.88^a^	30.54	476.19^a^	35.86	339.26^b^	14.69	327.24^b^	19.63	NS	**	NS
d 31 to 60	829.06^a^	43.97	869.89^a^	57.52	727.96^b^	39.95	763.75^b^	45.13	NS	**	NS
d 61 to 90	926.19^a^	50.24	918.69^a^	35.69	736.60^c^	33.38	812.31^b^	44.15	NS	*	NS
d 91 to 120	881.25	64.96	909.35	76.08	763.63	61.77	864.33	33.08	NS	**	NS
d 121 to 150	814.83	79.96	825.38	41.05	637.42	21.15	849.04	64.89	NS	NS	NS
Feed/gain ratio											
d 1 to 30	1.89^b^	0.07	1.78^b^	0.09	2.20^a^	0.07	2.03^ab^	0.11	0.056	**	NS
d 31 to 60	2.16^ab^	0.06	2.07^ab^	0.10	2.20^a^	0.07	1.92^b^	0.09	*	NS	NS
d 61 to 90	2.82^ab^	0.18	2.36^b^	0.09	2.90^a^	0.14	2.52^b^	0.14	**	NS	NS
d 91 to 120	3.03	0.16	2.70	0.19	3.15	0.41	2.48	0.12	0.09	NS	NS
d 121 to 150	3.68	0.42	3.13	0.21	3.73	0.19	2.94	0.19	NS	NS	NS

NBW, normal birth weight; LBW, low birth weight; NND, normal nutrient dense diet, HND, high nutrient dense diet. ^a,b^Denotes *P* < 0.05, *denotes *P* < 0.05, **denotes *P* < 0.01, NS, *P* > 0.05.

### Circulating metabolites and hormone secretions

As shown in Table 3, the circulating concentrations of triglycerides were greater in pigs fed the HND diet than that of pigs fed the NND diet at d 30, 60 and 90 of the experiment (*P* < 0.05). Birth weight affected the circulating triglyceride concentrations at d 60 and 150 of the experiment (*P* < 0.05). The total cholesterol concentrations were elevated at d 120 of the experiment by the HND diet compared with the NND diet (*P* < 0.05). Birth weight affected the total cholesterol concentrations at d 30 and 90 of the experiment (*P* < 0.05). The circulating triglyceride and total cholesterol concentrations were not affected by the interaction between diet and birth weight at different experimental diets (*P* > 0.05).

**Table 3:** Influence of birth weight and diet on circulating concentrations of metabolites and leptinin pigs.

	NBW				LBW				Significance		
	NND		HND		NND		HND		Diet	Birth weight	Interaction
	Mean	SEM	Mean	SEM	Mean	SEM	Mean	SEM			
Triglycerides, mmol/l											
Initial	0.424	0.014	0.432	0.036	0.461	0.029	0.448	0.025	NS	NS	NS
d 30	0.451^a^	0.028	0.501^ab^	0.013	0.489^ab^	0.022	0.540^b^	0.030	*	NS	NS
d 60	0.434^a^	0.014	0.474^a^	0.025	0.460^a^	0.018	0.545^b^	0.015	**	*	NS
d 90	0.451^a^	0.026	0.517^ab^	0.024	0.509^ab^	0.027	0.548^b^	0.024	*	NS	NS
d 120	0.459	0.039	0.510	0.026	0.526	0.032	0.573	0.042	NS	NS	NS
d 150	0.453^a^	0.043	0.481^ab^	0.037	0.57^bc^	0.025	0.635^c^	0.043	NS	**	NS
Total cholesterol, mmol/l											
Initial	2.572	0.196	2.586	0.165	2.598	0.162	2.535	0.111	NS	NS	NS
d 30	2.871^a^	0.210	2.922^a^	0.109	3.109^ab^	0.155	3.392^b^	0.119	NS	*	NS
d 60	2.737	0.220	2.789	0.127	3.012	0.253	3.198	0.184	NS	NS	NS
d 90	2.699^a^	0.194	3.191^ab^	0.184	3.138^ab^	0.184	3.538^b^	0.235	0.059	*	NS
d 120	2.772^a^	0.280	2.850^a^	0.221	3.085^ab^	0.330	3.659^b^	0.232	*	NS	NS
d 150	2.989	0.295	3.484	0.262	3.261	0.276	3.735	0.403	NS	NS	NS
Leptin, ng/ml											
Initial	2.75	0.25	2.59	0.16	2.99	0.30	3.20	0.32	NS	NS	NS
d 30	2.63	0.36	2.51	0.30	2.55	0.27	2.65	0.33	NS	NS	NS
d 60	2.81	0.27	2.98	0.30	3.26	0.30	2.93	0.32	NS	NS	NS
d 90	2.76	0.39	2.94	0.44	2.92	0.32	2.78	0.37	NS	NS	NS
d 120	2.73	0.35	2.90	0.26	2.76	0.27	3.32	0.36	NS	NS	NS
d 150	2.95^a^	0.30	2.89^a^	0.28	2.68^a^	0.29	3.93^b^	0.32	NS	0.056	*

NBW, normal birth weight; LBW, low birth weight; NND, normal nutrient dense diet, HND, high nutrient dense diet. ^a,b^Denotes *P* < 0.05, *denotes *P* < 0.05, **denotes *P* < 0.01,NS, *P* > 0.05.

Leptin concentrations were not affected by diet, birth weight or the interaction between diet and birth weight at the beginning, d 30, 60, 90 and 120 of the experiment (*P* > 0.05; Table 3). However, leptin concentration was significantly affected by the interaction between diet and birth weight at d 150 of the experiment (*P* < 0.05; Table 3), and LBW pigs fed the HND diet had significantly greater leptin concentrations than the other treatment groups (*P* < 0.05; Table 3).

### Glucose tolerance test

**Figure 2:**
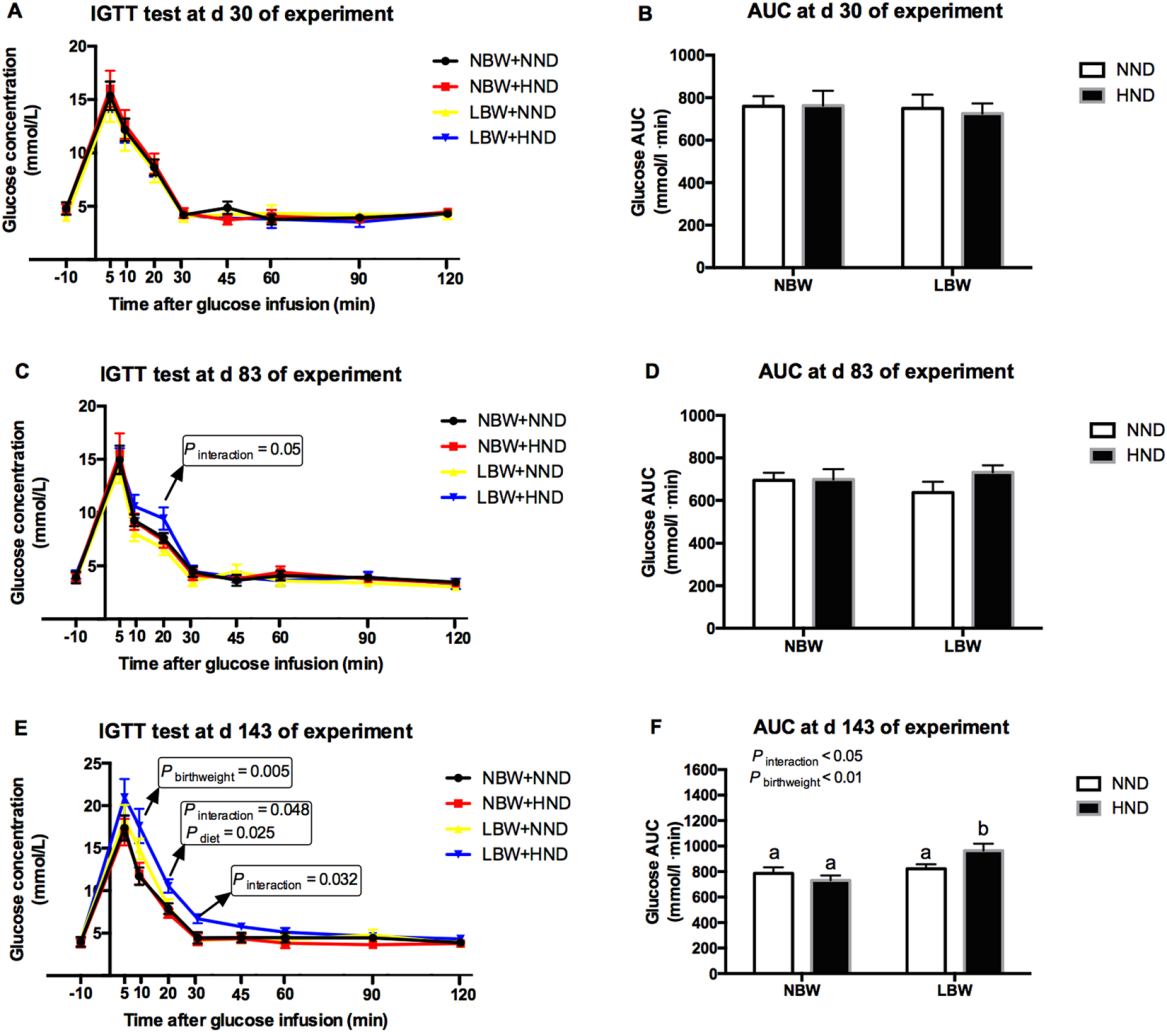
Intravenous glucose tolerance test (IGTT) of pigs fed different dense diets over time (n = 8). (**A**) IGTT at d 30 of the experiment; (**B**) Glucose area under curve (AUC) at d 30 of the experiment; (**C**) IGTT at d 83 of the experiment; (**D**) Glucose a AUC at d 83 of the experiment; (**E**) IGTT at d 143 of the experiment; (**D**) Glucose AUC at d 143 of the experiment. Column with different superscripts. ^a,b^Denotes *P* < 0.05. NBW, normal birth weight; LBW, low birth weight; NND, normal nutrient density; HND, high nutrient density.

As shown in Fig. 2, the IGTT revealed that the glucose tolerance was not affected at d 30 of the experiment (Fig. 2A and B). At day 83 of the experiment, the glucose concentration at 20 min after IGTT was affected by the interaction between birth weight and diet (*P* = 0.050, Fig. 2C), which was greater in the LBW pig fed the HND diet than that fed the NND diet (*P* < 0.05, Fig. 2C). Glucose AUC at day 83 of the experiment was not affected among different groups (*P* > 0.05, Fig. 2D). At d 143 of the experiment, the glucose concentration was affected at 10 min by birthweight (*P* = 0.005, Fig. 2E), at 20 min by diet (*P* = 0.025, Fig. 2E) and the interaction between birth weight and diet (*P* = 0.048, Fig. 2E), and at 30 min by the interaction between birth weight and diet (*P* = 0.032, Fig. 2E) after the IGTT. Glucose AUC at day 143 of the experiment was affected by birthweight (*P* < 0.01, Fig. 2F) and the interaction between birth weight and diet (*P* < 0.05, Fig. 2F). Notably, the glucose AUC of LBW pigs fed the HND diet was greater than that in the other groups (*P* < 0.05, Fig. 2F).

### Gene expressions

**Figure 3:**
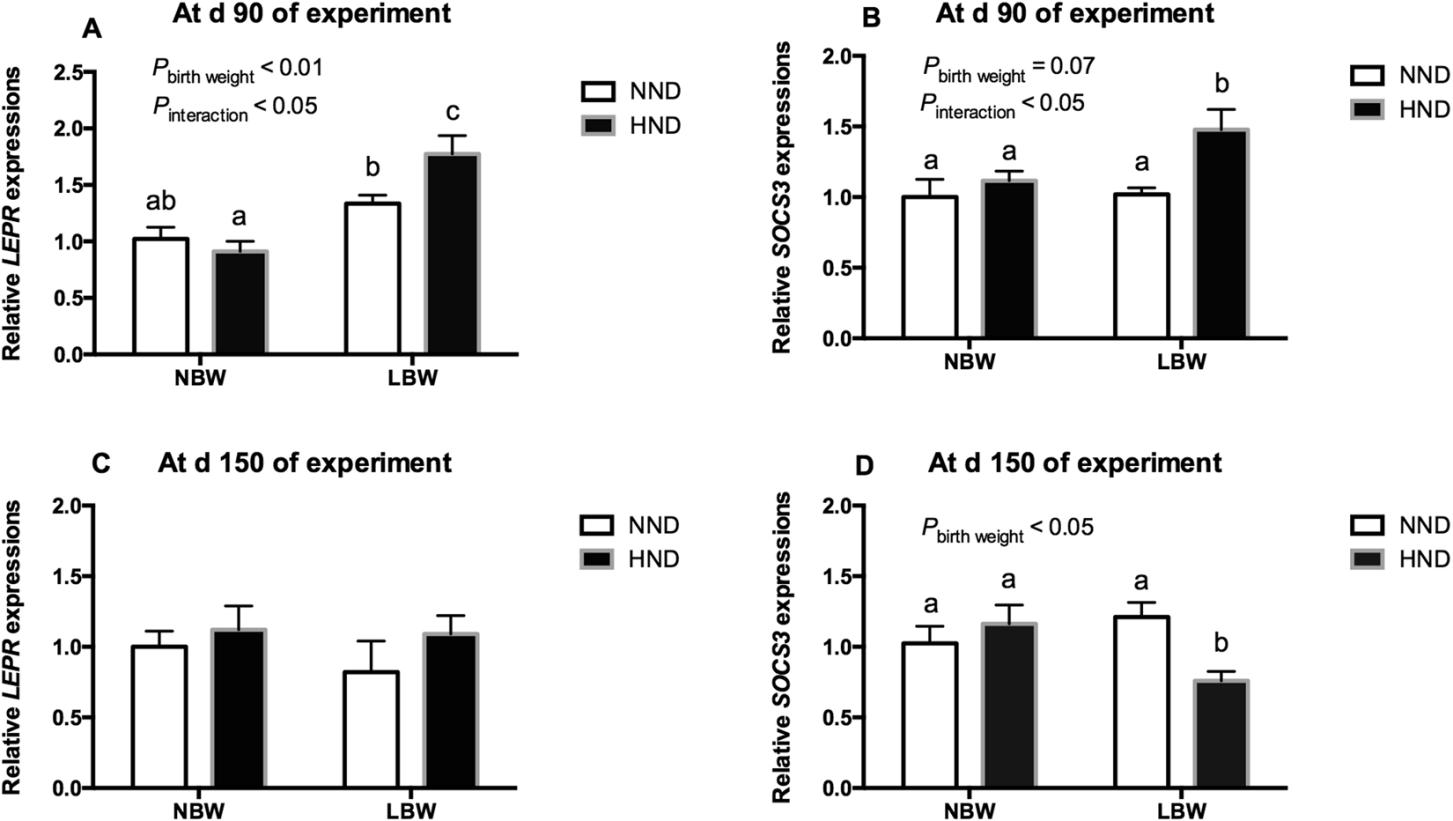
Influence of birth weight and diet on *LEPR* and *SOCS3* gene expression in the hypothalamic tissue over time (n = 8). (**A**) *LEPR* expression at d 90 of the experiment; (**B**) *SOCS3* expression at d 90 of the experiment; (**C**) LEPR expression at d 150 of the experiment; (**D**) *SOCS3* expression at d 150 of the experiment. Column with different superscripts. ^a,b^Denotes *P* < 0.05. NBW, normal birth weight; LBW, low birth weight; NND, normal nutrient density; HND, high nutrient density.

At d 90 of the experiment, mRNA expressions of *LEPR* in the hypothalamic tissue were affected by birth weight (*P* < 0.01, Fig. 3A) and the interaction between birth weight and diet (*P* < 0.05, Fig. 3A). The mRNA expressions of *LEPR* in the hypothalamus in LBW pigs fed the HND diet were significantly higher than that of pigs fed the NND diet (*P* < 0.05, Fig. 3A). The mRNA expressions in the hypothalamus of *SOCS3* were affected by an interaction between birth weight and diet (*P* < 0.05, Fig. 3B). While the HND diet did not affect *SOCS3* mRNA expression in NBW pigs (*P* > 0.05, Fig. 3B), the *SOCS3* mRNA expressions were up-regulated in LBW pigs by the HND diet compared with the other groups (*P* < 0.05, Fig. 3B). At d 150 of the experiment, *LEPR* mRNA expressions in the hypothalamic tissue were not affected by diet, birth weight or interaction between birth weight and diet (Fig. 3C). The expressions of *SOCS3* in the hypothalamus were affected by birth weight (*P* < 0.05, Fig. 3D). The HND diet did not affect *SOCS3* mRNA expression in NBW pigs (*P* > 0.05, Fig. 3D), but the *SOCS3* mRNA expression was down-regulated in LBW pigs by the HND diet compared with pigs fed the NND diets (*P* < 0.05, Fig. 3D).

**Figure 4:**
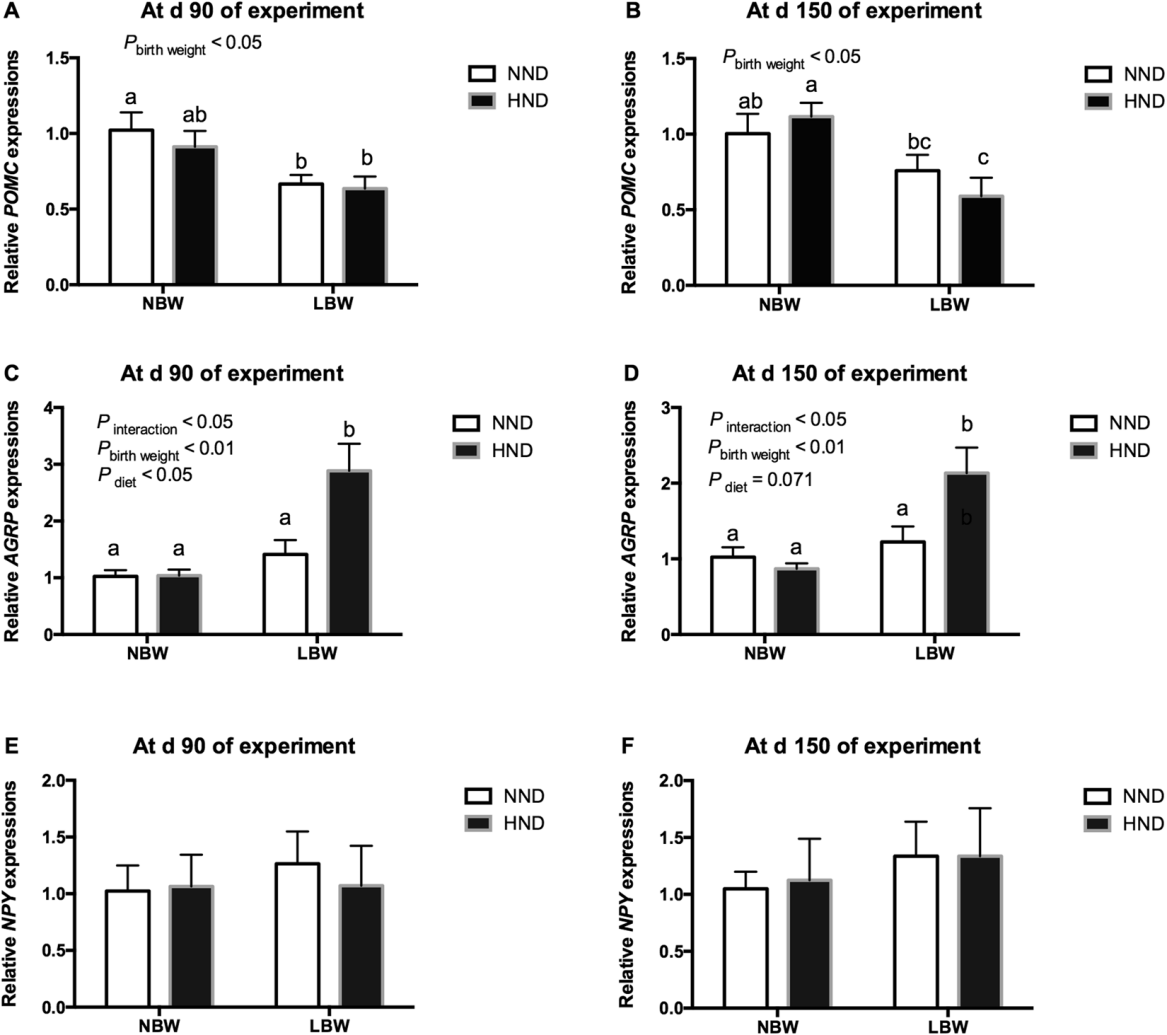
Influence of birth weight and diet on gene expressions of appetite regulatory peptides in the hypothalamic tissue over time (n = 8). (**A**) *POMC* gene expression at d 90 of the experiment; (**B**) *POMC* gene expression at d 150 of the experiment; (**C**) *AGRP* gene expression at d 90 of the experiment; (**D**) *AGRP* gene expression at d 150 of the experiment; (**E**) *NPY* gene expression at d 90 of the experiment; (**F**) *NPY* gene expression at d 150 of the experiment. Column with different superscripts. ^a,b^Denotes *P* < 0.05. NBW + NND, normal birth weight pigs fed the normal nutrient dense diet; NBW + HND, normal birth weight pigs fed the high nutrient dense diet; LBW + NND, low birth weight pigs fed the normal nutrient dense diet; LBW + HND, low birth weight pigs fed the high nutrient dense diet.

The mRNA expression level of anorexigenic *POMC* and orexigenic *NPY* and *AGRP* in hypothalamus were shown in Fig. 4. The mRNA expressions of POMC were decreased by low birthweight at d 90 and 150 of the experiment (*P* < 0.05, Fig. 4A and B). The mRNA expressions of *POMC* in hypothalamus were affected by birth weight (*P* < 0.01) or interaction between birth weight and diet (*P* < 0.05) at d 90 and 150 of the experiment (Fig. 4C and D). The *POMC* mRNA expression at d 90 of the experiment (*P* < 0.05, Fig. 4C), but not at d 150 of the experiment(*P* < 0.05, Fig. 4D), were affected by diets. Notably, the mRNA expression of *AGRP* was greater in LBW pigs fed the HND diet than the other groups (*P* < 0.05, Fig. 4C and D). The mRNA expressions of *NPY* in hypothalamus were not affected by diets, birth weight or interaction between birth weight and diet (*P* > 0.05, Fig. 4E and F).

**Figure 5:**
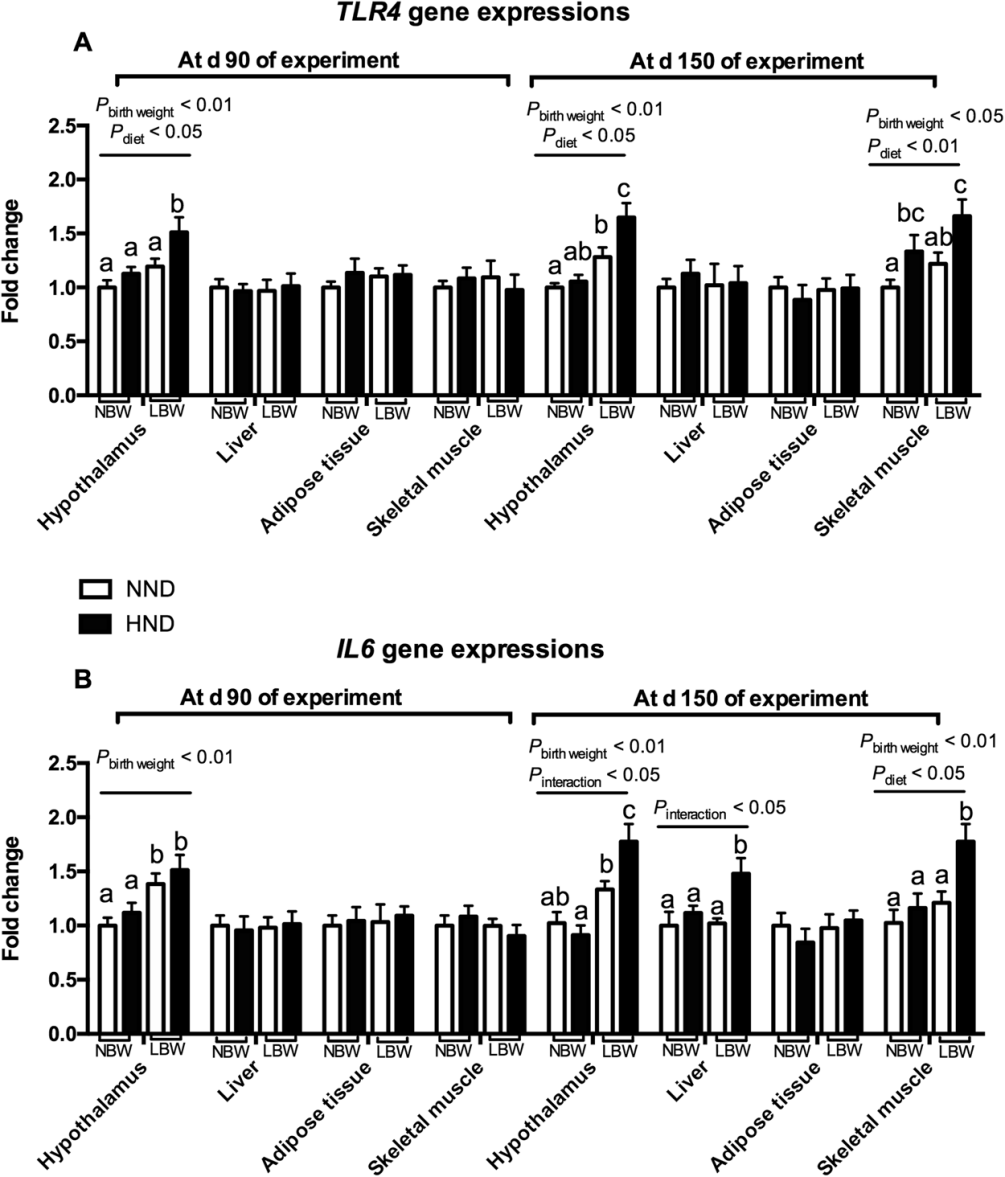
Influence of birth weight and diet on*TLR4 and IL6* gene expression in the hypothalamic tissue, liver, adipose tissue and skeletal muscle over time (n = 8). (**A**) *TLR4* gene expression at d 90 and 150 of the experiment; (**B**) *IL6* gene expression at d 90 and 150 of the experiment. Column with different superscripts. ^a,b,c^Denotes *P* < 0.05. NBW + NND, normal birth weight pigs fed the normal nutrient dense diet; NBW + HND, normal birth weight pigs fed the high nutrient dense diet; LBW + NND, low birth weight pigs fed the normal nutrient dense diet; LBW + HND, low birth weight pigs fed the high nutrient dense diet.

The mRNA expression of *TLR4* in the hypothalamus, but not in liver, adipose tissue or skeletal muscle, were affected by birth weight (*P* < 0.01, Fig. 5A) and diet (*P* < 0.05, Fig. 5A) at d 90 of the experiment. At d 150 of experiment, the mRNA expression of *TLR4* in the hypothalamus and skeletal muscle tissues were affected by birth weight and diet (*P* < 0.05 or *P* < 0.01, Fig. 5A). The mRNA expression of *IL6* in the hypothalamus, but not in the tissues collected from liver, adipose tissue or skeletal muscle, were affected by birth weight (*P* < 0.01, Fig. 5B) at d 90 of the experiment. At d 150 of the experiment, the mRNA expression of *TLR4* in the hypothalamus, liver and skeletal muscle tissues were affected by birth weight and diet (*P* < 0.05 or *P* < 0.01, Fig. 5B). The LBW pigs fed the HND diet had greater *TLR4* and *IL6* gene expression levels in the hypothalamus, liver and skeletal muscle than the other groups (*P* < 0.05).

### Protein expression

**Figure 6:**
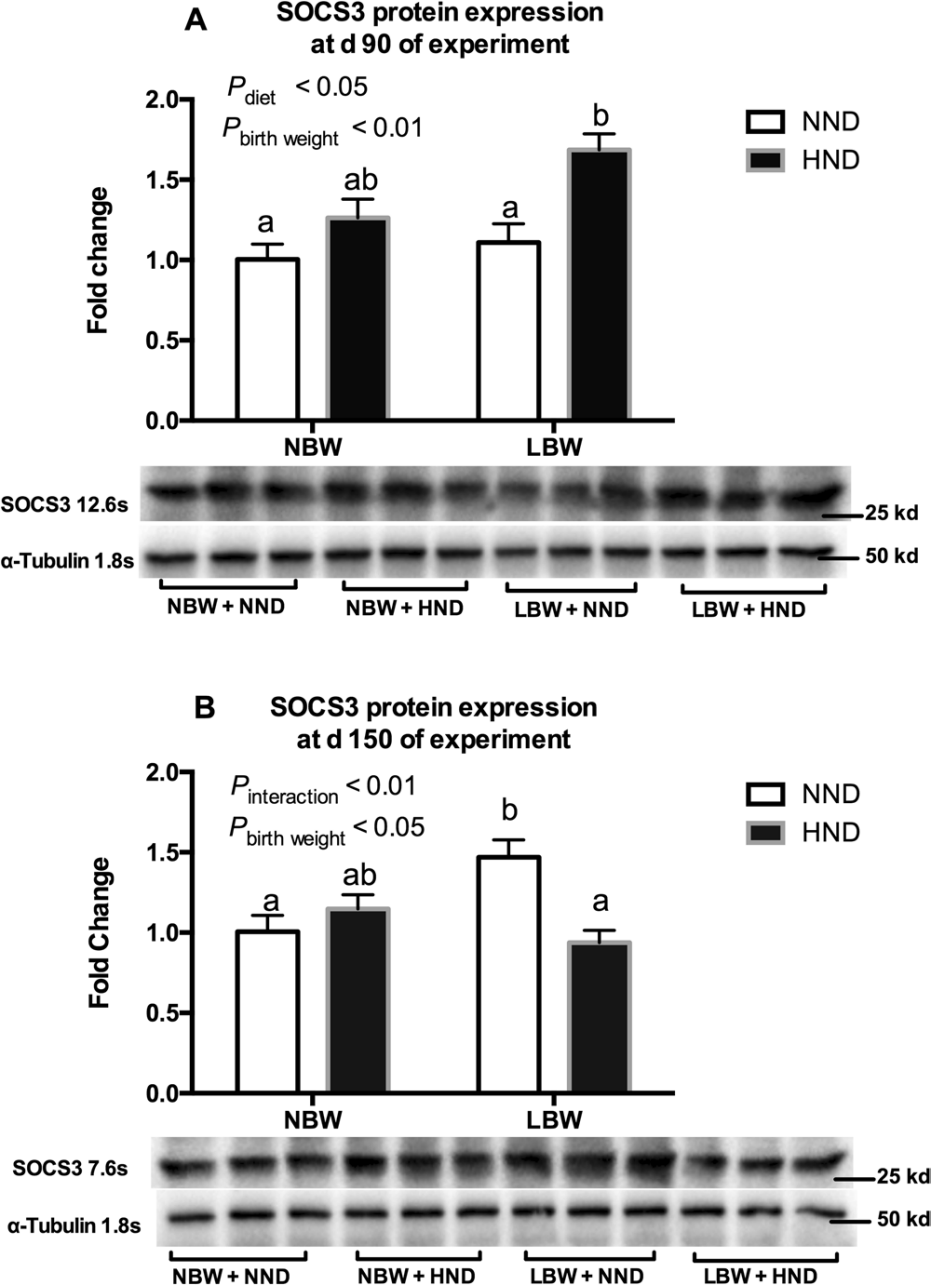
Influence of birth weight and diet on SOCS3 protein expression in the hypothalamic tissue over time (n = 6). (**A**) SOCS3 protein expression at d 90 of the experiment; (**B**) SOCS3 protein expression at d 150 of the experiment. Column with different superscripts. ^a,b^Denotes *P* < 0.05. NBW, normal birth weight; LBW, low birth weight; NND, normal nutrient density; HND, high nutrient density.

The protein expressions of SOCS3 were presented in Fig. 6 and Supplementary Fig. S1. The SOCS3 protein expression in the hypothalamus were significantly affected by diet (*P* < 0.05; Fig. 6A) and birth weight (*P* < 0.01; Fig. 6A) at d 90 of the experiment. The HND diet did not affect SOCS3 protein expression in NBW pigs (*P* > 0.05; Fig. 6A), but the SOCS3 protein expression was up-regulated in LBW pigs by the HND diet compared with pigs fed the NND diets (*P* < 0.05; Fig. 6A). At d 150 of the experiment, SOCS3 protein expression in the hypothalamus were significantly affected by an interaction between diet and birth weight (*P* < 0.01; Fig. 6B), and the SOCS3 protein expression were greater in LBW pigs fed the NND diet compared with the other groups (*P* < 0.05; Fig. 6B).

**Figure 7:**
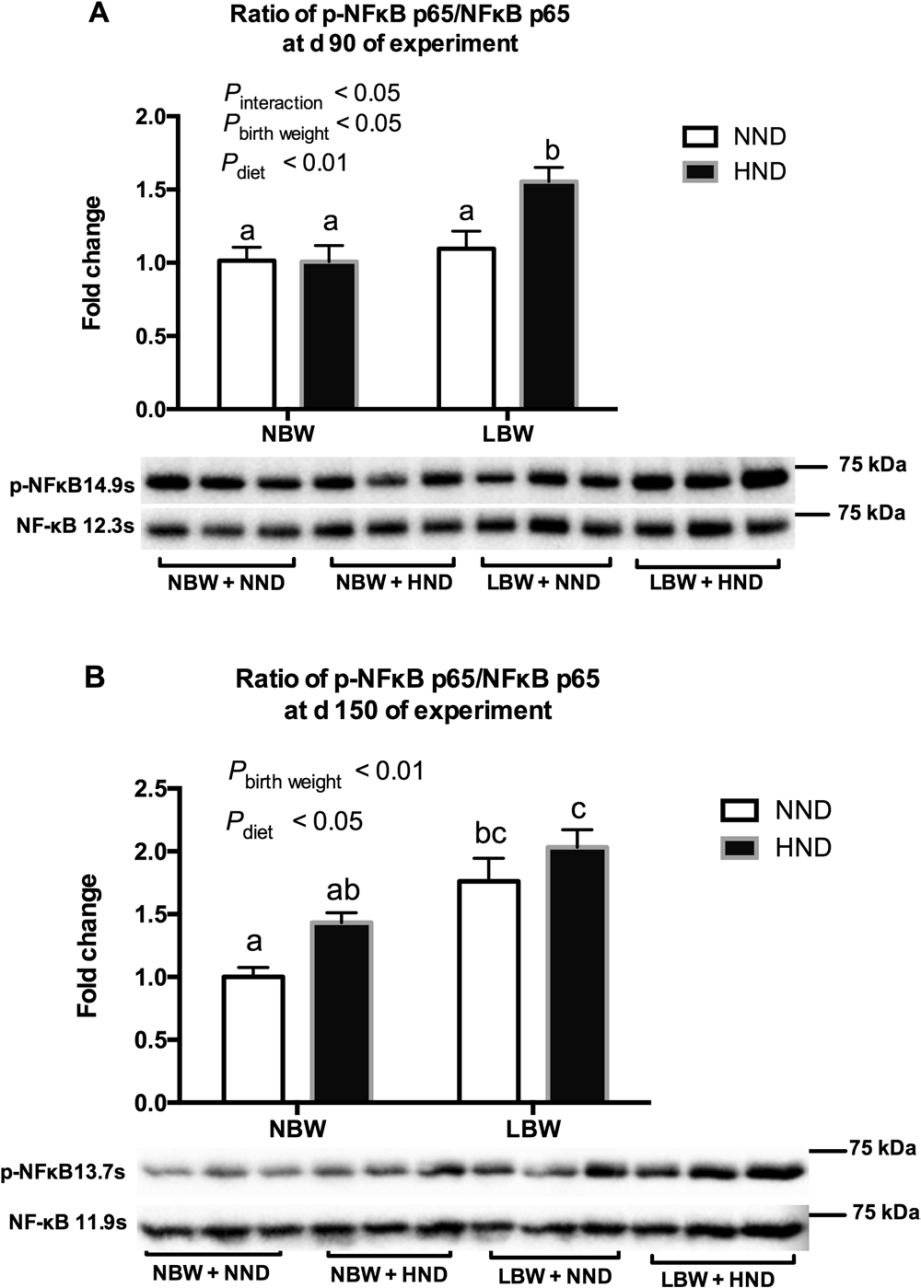
Influence of birth weight and diet on protein expressions of NFκB p65 and its phosphorylation in the hypothalamic tissue over time (n = 6). (**A**) Protein expressions of NFκB p65 and its phosphorylation at d 90 of the experiment; (**B**) Protein expressions of NFκB p65 and its phosphorylation at d 150 of the experiment. Column with different superscripts. ^a,b^Denotes *P* < 0.05. NBW, normal birth weight; LBW, low birth weight; NND, normal nutrient density; HND, high nutrient density.

The protein expressions of NFκB and its phosphorylation were presented in Fig. 7 and Supplementary Fig. S2. The ratio of p-NFκB p65 to NFκB p65 were affected by diet (*P* < 0.01), birth weight (*P* < 0.05) and an interaction between diet and birth weight (*P* < 0.05) at d 90 of the experiment (Fig. 7A), which were greater in LBW pigs fed the HND diet than the other groups (*P* < 0.05, Fig. 7A). The ratio of p-NFκB p65 to NFκB p65 expressions were affected by birthweight (*P* < 0.01; Fig. 7B) and diet (*P* < 0.05; Fig. 7B) at d 150 of the experiment.

## Discussion

In the present study, the LBW pigs did not achieve similar body weight as NBW pigs, which did not agree with previous researchers who reported that pigs born with LBW were able to reach similar body weights at adulthood^14,28,29^. The reason might be due to the difference of dietary energy intake between studies. Madsen and colleague^29^ found that the absolute energy intake between LBW and NBW pigs were similar during different period of experiment, resulting in greater daily weight gain. However, in the present study, the energy intake was lower for LBW pigs than NBW pigs before d 60 of the experiment. Body weight gain was not affected by the HND diet in pigs born with NBW, however, from d 121 to 150 of the experiment, body weight was greater in LBW pigs fed the HND diet compared with those fed the NND diet, suggesting that pigs born with varied body weight had differential responses to dietary nutrient density.

The LBW pigs had a lower feed intake and energy consumption than their NBW counterparts, however, when the feed and energy intake were calculated on a body weight basis, the relative feed intake and energy consumption were greater in LBW pigs than that in NBW pigs, and the HND diet amplified this difference among different treatment groups. Therefore, this greater amount of nutrient intake allows a possible higher accretion of body tissue and forms a basis for catch-up growth of the LBW pigs. This might be the reason why LBW pigs fed the HND diet have the lowest feed-to-gain ratio among the four dietary groups. The energy intake of NBW pigs was similar between the HND group and the NND group. However, the energy intake and the relative energy intake were greater in LBW pigs fed the HND diet compared with the NND diet, suggesting that LBW pigs have a distinct pattern of mechanisms controlling energy-intake behavior.

The greater energy intake of LBW individual might be due to alternation of expression of appetite regulatory peptides^30^. The neurons expressing NPY and AGRP provide an orexigenic drive while neurons expressing POMC are anorexigenic. In the present study, AGRP was up-regulated and POMC was down-regulated in LBW pigs than the NBW pigs, suggesting that the LBW individual might be born with a distinct pattern of a feeding control mechanism^30^. Early research revealed that IUGR individual exhibit different hypothalamic distribution of leptin receptors that may be linked to postnatal altered feeding behavior and energy metabolism^14,31^. The hypothalamic expression of *LEPR* in the hypothalamus was detected in pigs fed the NND or the HND diets, and results revealed that *LEPR* expression in the hypothalamus were significantly higher in LBW pigs compared with NBW pigs. Furthermore, the HND diet elevated the expression of *LEPR* in the hypothalamus to a greater extent than the NND diet fed to LBW pigs at d 90 of the experiment. The greater *LEPR* expression in the hypothalamus of LBW pigs was not consistent with previous research which found that LBW pigs had a lower number of LEPR-expressing neurons in the hypothalamic arcuate nucleus^13^. The reason for this inconsistency could be attributed to differences in the age of animals used between studies.

The alternation of hypothalamic *LEPR* expression implies that the leptin signaling was changed in LBW pigs fed diets of different nutrient density. When leptin binds to its receptor, one of the crucial pathways activated is the janus kinase signaling transduction/transcription activator (JAK2–STAT3) cascade^32^. As a direct transcriptional product of STAT3, SOCS3 is commonly thought to play a pathophysiological role in obesity-associated leptin resistance^33^. Concurrent with the greater mRNA expression of *LEPR*, the *SOCS3* mRNA and its protein expression were significantly higher in LBW pigs fed the HND diet at d 90 of the experiment, suggesting that leptin signaling was effectively elevated in LBW pigs fed the HND diet. However, the expression patterns of *LEPR* and *SOCS3* at d 150 of experiment were different from that at d 90 of experiment. The *LEPR* expression was similar among different groups, but the *SOCS3* mRNA and its protein expression in LBW pigs fed a HND diet was significantly lower than the other groups at d 150 of the experiment. This might be attributed to the fact that SOCS3 can inhibit leptin signaling^33^, which could explain why intake of energy was similar before the d 90 of experiment, but was greater for LBW pigs fed the HND diet compared with the NND diet at d 150 of the experiment.

Leptin, an adipocyte-derived hormone that can enter the brain to regulate food intake and energy expenditure, was greater in the circulation of LBW pigs fed the HND diet than the other groups. Together with the greater energy intake, the LBW pigs fed the HND diet showed symptoms of leptin resistance. The decreased *SOCS3* gene and protein expression might play a role in this process based on the previous research which showed that knockdown of SOCS3 in the hypothalamus could induce leptin resistance and alter energy balance^34,35^.

The chronic inflammatory response has been recognized as a common cause that can induce dysregulated energy balance and metabolic diseases^36^, thus, the pro-inflammatory *TLR4* and *IL6* gene expression were investigated in the hypothalamus, liver, adipose, and skeletal muscle. Interestingly, the hypothalamic *TLR4* and *IL6* mRNA expression changed faster in pigs by birth weight and diets, and LBW pigs fed the HND diet had a significantly greater *TLR4* and *IL6* mRNA expressions than the other groups at d 90 or d 150 of the experiment, strongly implicating that the hypothalamic inflammatory response was involved in the changes of feeding behavior of LBW pigs fed the HND diet. Additionally, the protein expressions of NFκB p65 and its phosphorylation, which play a pivotal role in inflammatory and immune responses, was elevated in LBW pigs fed the HND diets, further confirming that the LBW pigs had a greater inflammatory response to a HND diet.

Recently, hypothalamic inflammation has been proposed as a novel underlying mechanism regulating arcuate neuronal activity and feeding behavior^36,37^, and SOCS3 was observed to play an important role in the regulation of infection and inflammation^38^. In line with the early hypothalamic inflammation response, the IGTT test also revealed that LBW pigs fed the HND diet showed greater glucose intolerance than the other groups as early as d 83 of the experiment, which was in agreement with previous research who showed that the inflammation of hypothalamus can be activated under obesogenic condition to promote energy, body weight and glucose imbalance^39,40^. These evidence, together with the results of this study, imply that LEPR and SOCS3 signaling coordinate with the hypothalamic inflammatory response to alter the feeding behavior and the postnatal glucose disposal of LBW pigs fed diets of different nutrient density.

## Conclusion

Collectively, these results demonstrate that, compared to normal birth weight pigs, low birth weight pigs had a different hypothalamic leptin and inflammatory response to a high nutrient dense diet, which contributed to greater energy intake and glucose intolerance.

## Electronic supplementary material

Supplementary Information
