# Supplementary material for: A high nutrient dense diet alters hypothalamic gene expressions to influence energy intake in pigs born with low birth weight
Source: Sci Rep. 2018 Apr 3;8:5514. doi: 10.1038/s41598-018-23926-x (PMC5882958; doi:10.1038/s41598-018-23926-x)
Supplement: Supplementary file 1 — Supplementary Information [file 41598_2018_23926_MOESM1_ESM.pdf]

# A high nutrient dense diet alters hypothalamic gene expressions to influence energy intake in pigs born with low birth weight

Jingbo Liu[1,2]*, Shanchuan Cao[1,2], Ming Liu[1], Liang Chen[1], Hongfu Zhang[1,]*

*[1]State Key Laboratory of Animal Nutrition, Institute of Animal Sciences, Chinese Academy of Agricultural Sciences, Beijing 100193, P. R. China*
*[2]School of Life Science and Engineering, Southwest University of Science and Technology, Mianyang 621010, Sichuan, P. R. China*

*Corresponding author,

Jingbo Liu,

Tel: 86 13618105888

Fax: 86 0816-6089529

Email: liuswust@163.com

And Hongfu Zhang,

Tel: 86 010-62816249

Fax: 86 010-62818910

Email: zhanghongfu@caas.cn

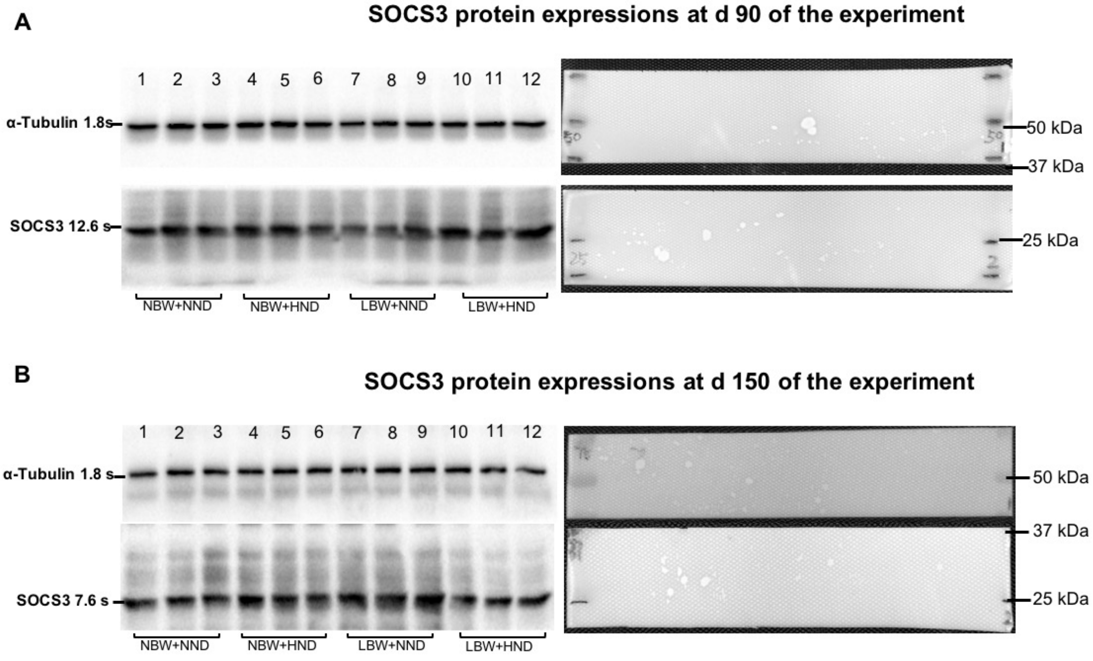


**Supplementary Figure S1.** SOCS3 protein expressions at d 90 (A) and d 150 (B) of the experiment. Twelve hypothalamic samples (n = 3 per group) were loaded from lane 1 to 12. Protein samples, 30 μg per lane, were loaded in each lane. One PVDF membrane was cut into two pieces at the ladder 37 and were incubated with anti-$\alpha$-tubulin (predicted molecular weight at 52 kDa) or anti-SOCS3 (predicted molecular weight at 27 kDa) antibodies, respectively. NBW, normal birth weight; LBW, low birth weight; NND, normal nutrient density; HND, high nutrient density.

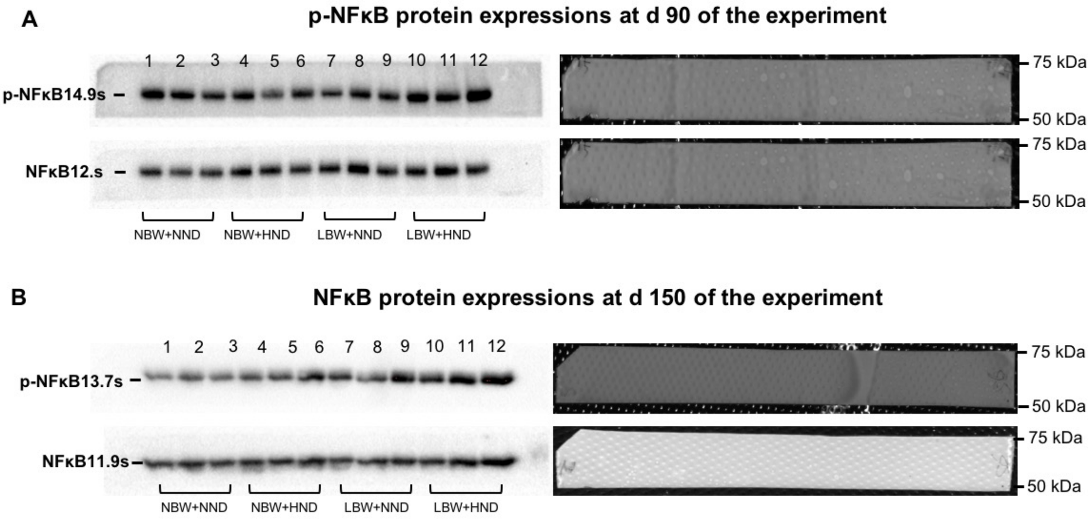


**Supplementary Figure S2.** NFκB protein expressions at d 90 (A) and d 150 (B) of the experiment. Twelve hypothalamic samples (n = 3 per group) were loaded from lane 1 to 12. Protein samples, 30 μg per lane, were loaded in each lane. One piece of PVDF membrane was cut between the ladder 50 and 75 kDa, and were incubated with antibodies against p-NFκB (predicted molecular weight at 65 kDa). After the detection of p-NFκB, the same membrane were washed with stripping buffer (#P0025, Byotime, Shanghai, China) for 15 min and were then incubated with antibody against NFκB. NBW, normal birth weight; LBW, low birth weight; NND, normal nutrient density; HND, high nutrient density.
